# Exploring association between *MBL2* gene polymorphisms and the occurrence of clinical blackwater fever through a case–control study in Congolese children

**DOI:** 10.1186/s12936-020-3100-8

**Published:** 2020-01-15

**Authors:** Joseph M. Bodi, Célestin N. Nsibu, Roland L. Longenge, Michel N. Aloni, Pierre Z. Akilimali, Patrick K. Kayembe, Ahmeddin H. Omar, Jan Verhaegen, Pierre M. Tshibassu, Prosper T. Lukusa, Aimé Lumaka, Kenji Hirayama

**Affiliations:** 1 Department of Pediatrics, Emergency and Intensive Care Unit, University Hospital of Kinshasa, Faculty of Medicine, University of Kinshasa, Kinshasa, Democratic Republic of Congo; 2 Department of Pediatrics, Haemato-oncology and Nephrology Unit, University Hospital of Kinshasa, Faculty of Medicine, University of Kinshasa, Kinshasa, Democratic Republic of Congo; 3 Division of Biostatistics and Epidemiology, School of Public Health, University of Kinshasa, Kinshasa, Democratic Republic of Congo; 4 Division of Malaria Control (DOMC), Ministry of Health, Nairobi, Kenya; 5 Department of Microbiology, Katholieke Universiteit de Leuven, Brussels, Belgium; 6 Department of Pediatrics, Gastroenterology and Neurology Unit, University Hospital of Kinshasa, Faculty of Medicine, University of Kinshasa, Kinshasa, Democratic Republic of Congo; 7 Center for Human Genetics, Department of Pediatrics, Faculty of Medicine, University of Kinshasa, Kinshasa, Democratic Republic of Congo; 8 Center for Human Genetics, Department of Pediatrics, Faculty of Medicine, University of Kinshasa, Kinshasa, Democratic Republic of Congo; 9 Department of Immunogenetics, Institute of Tropical Medicine (Nekken), University of Nagasaki, Nagasaki, Japan

**Keywords:** Mannose-Binding Lectin 2, *MBL2*, Blackwater fever, Severe malaria, Democratic Republic of Congo

## Abstract

**Background:**

Blackwater fever (BWF), one of the most severe and life-threatening forms of falciparum malaria, is characterized by acute massive intravascular haemolysis, often leading to acute renal failure. Thus far, the genetics of the underlying susceptibility to develop BWF is not fully elucidated. Deficiency in the MBL protein, an important component of the innate immune system, has previously been suggested to be a susceptibility factor for the development of severe malaria. This study aimed to evaluate the association between *MBL2* gene polymorphisms, known to affect the MBL protein level/activity, and the occurrence of BWF among Congolese children.

**Methods:**

This is a case–control study. Cases were patients with BWF, whereas controls, matched for gender and age, had uncomplicated malaria (UM). Dried blood spot was collected for genotyping.

**Results:**

A total of 129 children were screened, including 43 BWF and 86 UM. The common allele in BWF and UM was A, with a frequency of 76.7 and 61.0%, respectively (OR: 2.67 (0.87–829) and *p *= 0.079). The frequency of the C allele was 18.6 and 29.1% in BWF and UM groups, respectively, with *p *= 0.858. Not a single D allele was encountered. Genotype AA was at higher risk for BWF whereas genotypes A0 (AB and AC) were over-represented in UM group (OR: 0.21 (0.06–0.78)) with *p *= 0.019. Nine haplotypes were observed in this study: 3 high MBL expression haplotypes and 6 low MBL expression haplotype. One new haplotype HYPC was observed in this study. None of these haplotypes was significantly associated with BWF.

**Conclusion:**

This pilot study is a preliminary research on *MBL2* gene and infectious diseases in DRC. The study results show a higher risk for BWF in AA. This suggests that future studies on BWF should further investigate the contribution of a strong immune response to the occurrence of BWF.

## Background

Mannose-Binding Lectin protein (MBL), encoded by *MBL2* gene (Mannose-Binding Lectin soluble 2; OMIM: 154545), is an important component of the innate immune system with 4 main functions, including activation of complement, direct promotion of opsono-phagocytosis, modulation of the inflammatory response, and promotion of apoptosis [1]. There are also other promoter variants that may affect gene expression [2–5]. The MBL deficiency, also known as ‘dysfunctional MBL’, is one of the most common immune deficiencies in the world [2]. Three non-synonymous single nucleotide substitutions in the exon 1 of *MBL2* gene cause dramatic decrease of MBL in heterozygous state or almost complete absence of MBL in homozygous or compound heterozygous state. These include substitutions at codon 52 (CGT =>TGT; p.Arg52Cys, rs5030737), codon 54 (GGC ≥ GAC; p.Gly54Asp, rs1800450) and codon 57 (GCA ≥ GAA, p.Gly57Glu, rs1800451) [6–9]. Based on the classic *MBL2* polymorphisms codification, substitutions at codons 52, 54 and 57 are referred to as *D, B* and *C* derived alleles, respectively, whereas the ancestral allele is known as allele A  [10]. Because these three variant alleles cause similar MBL deficiency, the concept of ‘*O*’ allele is used to describe either of these variants [8].

In addition, 3 substitutions including 2 in the promoter region of *MBL2* (-550C/G or rs11003125 and -221G/C or rs7096206) and one in the UTR within the exon 1 (c.4T/C or rs7095891) have been shown to affect the level of MBL protein and influence the outcome of infectious diseases [9, 10]. The derived alleles in the promoter region, the upstream region and the exon 1 have been previously combined into haplotypes [10]. The *MBL2* haplotypes *HYPA, LYQA, LYPA,* and *HYQA* have been associated with high *MBL2* expression. Conversely, haplotypes *LXPA, LYPB, LYQC, HYPD, LYPD, HYQC, LXPB,* and *LYQB* showed low *MBL2* expression [10]. However, a recent haplotype, termed HYPC, was identified in similar sub-Saharan individuals in a study from Zimbabwe [11].

In the Democratic Republic of Congo (DRC), *Plasmodium falciparum* is the most severe and lethal species of malaria parasite among children below 5 years of age [12–15]. The clinical expression of falciparum malaria consists of a wide spectrum, spanning from asymptomatically infected to multiple severe forms depending on multiple factors [16]. Blackwater fever (BWF), one of the life-threatening forms of falciparum malaria, is characterized by acute massive intravascular haemolysis and, usually, acute renal failure which occurs after using quinine in the treatment of malaria [17–21]. Factors such as inadequate malarial immunity, misuse of quinine and *G6PD*-deficiency have been associated with the occurrence of BWF [22–26]. However, the underlying genetics of the susceptibility to develop BWF is not fully elucidated.

Two apparently contradictory theories are proposed to explain the involvement of MBL in severe forms of infections such as malaria. On one hand, MBL deficiency is known to be a susceptibility factor for the development of severe infections including malaria [23, 24, 26–33]. On the other hand, MBL deficiency is also thought to be protective against certain complications by preventing excessive activation of the immune response, avoiding thereby deleterious immune-related complications during infections [7, 34, 35]. It has been recently reported that malaria IgG are significantly elevated in BWF [36], which also suggests that unlike other severe forms of malaria, BWF would more likely occur in normal or hyper immune individuals. A straight connection between IgG antibodies and *MBL2* alleles have been established in a study on *Chlamydia pneumoniae* where the mean antibody titre increases with the number of copies of ancestral *MBL2* alleles [37]. Although it remains unclear how ancestral *MBL2* variants increase antibody titres and whether this matches with known mechanisms of MBL in the immune response, it could be hypothesized that unlike in other severe forms of malaria, people with ancestral *MBL2* alleles would be at higher risk to exhibit BWF.

To date, the distribution of *MBL*2 alleles and their possible association to BWF in the DRC have not been investigated.

## Methods

### Study aims, design and setting

This study aimed to test the association between *MBL2* polymorphisms and Blackwater fever, one of the most severe complications of malaria, and provide the first distribution data for *MBL2* haplotypes in Congolese individuals. This is a case–control study conducted over 2 years in 4 medical institutions across Kinshasa, namely University Hospitals of Kinshasa, Kimbanseke Hospital, Bondeko Hospital and General Provincial Hospital of Kinshasa. Sampling methods and case definition are published elsewhere [12]. Altogether, 43 cases and 86 controls were enrolled. Ages for cases and controls ranged from 2 to 15 years.

### Clinical evaluation

The medical history was obtained from parents, with particular attention to demographic data, including disease history and medications taken before BWF episode. Clinical data were recorded in a customized pre-tested clinical form. Malaria was confirmed by the presence of parasites on blood thick and film.

### Laboratory measurements

Twenty mL of fresh urine were collected from each participant. The presence of haemoglobin in urine was first detected by urinary dip strip (Medi test Combi9, MacheryEur, Paris, France) and then confirmed by spectrometer (Thermo Genesis 10 BIO, New York, USA) using protocol of 3,3′ dimethyl benzidine reagent [38]. The results of urine dip stick were read as either negative (yellow colour) or positive (change in blue colour) 1+, 2+, 3+, which corresponded approximately to haemoglobin concentrations of respectively 0.061 ± 0.0166 mg/L, 0.3986 ± 0.2612 mg/dL and 0.5679 ± 0.27688 mg/L as quantified using the spectrometer.

### DNA extraction and *MBL2* genotyping

Human *MBL2* gene was assessed from genomic DNA. Eight drops of blood were collected on FTA card^®^ WB 120067 (GE Healthcare, Amersham, UK) and stored in a fridge until the transfer to the Institute of Tropical Medicine of Nagasaki University in Japan for DNA testing in the Department of Immunogenetics according to a previously described protocol [39].

DNA samples from 43 cases and 86 controls were examined. The promoter region and exon 1 of *MBL2* gene were PCR-amplified and Sanger sequenced. Prior to Sanger sequencing, PCR products were verified by gel electrophoresis to confirm the presence of expected band and exclude unexpected inserts. The PCR mixture contained 17.5 μL of ultra-pure water, 2.5 μL PCR 10 × buffer, 4 μL of dNTPs (2 µmol), 0.4 μL (2 units) of Taq polymerase and 0.8 μL of each primer (2.5 µmol). A disc containing between 5 and 20 ng of DNA was punched from the FTA card and added into the PCR reaction tube. In order to identify technical contaminations, a tube a No DNA template was also included in each run. This consisted of a punch from an unspotted FTA card. After an initial denaturation step of 5 min at 95 °C, 35 amplification cycles were applied including rapid denaturation at 95 °C for 1 min, annealing at 65 °C for 1 min and elongation at 72 °C for 1 min. The reaction ended with a final elongation step at 72 °C for 5 min. PCR product was sequenced by dideoxy termination sequencing using Big-Dye^®^ Terminator version 1.1. Sequencing product was analysed on a 3730 DNA ANALYSER, version 3.0, from HITACHI. Haplotypes were double- and triple-checked using visual inspection of sequencing traces.

Alleles were designated as suggested by Antonarakis et al. [7] for the 3 variants in the Exon 1. The MBL2*B, MBL2*C and other variants alleles were identified as described by Sumiya et al., Lipscombe et al. and Madsen et al. [3–5, 9].

### Data management and analysis

Alleles and genotypes frequencies were obtained by direct scoring of electropherogram. Data were recorded using the software Epi Info 7. All analyses were carried out using SPSS 18.0. All records were crosschecked with the original data sheets before the analysis. A non-conditional model was used. This was a binary logistic regression including covariates, anti-malaria drugs, *MBL2* gene polymorphism, G6PD and parasitaemia. Multivariate logistic regression analysis was used to evaluate associations between *MBL2* haplotypes/genotypes/alleles and the BWF. Odds ratio and confidence intervals were calculated. All tests were two-sided, and the level of significance was set at *p *< 0.05.

## Results

A total of 129 Congolese children were investigated, including 43 cases and 86 controls. Sixty-eight were girls (52.7%) and 61 boys (47.3%). The mean age was 8.75 ± 3.73 years for all the study population, 8.62 ± 3.84 years and 8.55 ± 3.77 years, respectively, for cases and controls (uncomplicated malaria, UM), only 8 cases (18.6%) were below 5 years, which is the most vulnerable period for severe malaria, versus 20 patients (23.26%) in the control group. The majority of BWF cases (38 cases) occurred during the rainy season (88.4%) and 5 (11.6%) occurred during the dry season. Low parasitaemia was associated to BWF OR: 3.31 (1.41–7.79) with p = 0.005 (Table 1).

**Table 1:** Socio-demographic features of patients in the study population

	Case (n = 43)	Control (n = 86)	Total (n = 129)	OR (IC 95%)	p
Distribution for age					
≤ 5 years	8 (18.6)	20 (23.3)	28 (21.7)	1	
> 5 years	35 (81.4)	66 (76.7)	101 (78.3)	1.33 (0.53–3.32)	0.676
Sex (%)					
Male	21 (48.8)	40 (46.7)	61 (47.3)	1.10 (0.53–2.28)	0.803
Female	22 (51.2)	46 (53.5)	68 (52.7)	1	
Season					
Rainy	38 (88.4)	51 (59.3)	89 (69.0)	5.22 (1.87–14.56)	< 0.001
Dry	5 (11.6)	35 (40.7)	40 (31.0)	1	
*Plasmodium*					
*Falciparum*	37 (86.0)	73 (84.9)	110 (85.3)	1.10 (0.39–3.12)	0.860
*Falciparum*–*malariae*	6 (14.0)	13 (15.1)	19 (14.7)	1	
Parasitaemia (parasites/µl)					
Low (< 1000 tropho/µl)	33 (76.7)	43 (51.8)	76 (61.3)	3.31 (1.41–7.78)	0.005
High (≥ 1000 tropho/µl)	10 (23.2)	40 (48.2)	48 (38.7)	1	

Using a non-conditional model, a binary logistic regression, including covariant, anti-malaria drugs, *MBL2* gene polymorphism, G6PD and parasitaemia, it was observed that MBL2*AB or AC is protective factor in the development of BWF. OR: 0.09 (0.01–0.63), with p = 0.015. The association with quinine intake and low parasitaemia, observed in this study (Table 2), was already published [12].

**Table 2:** Determinant factors of Blackwater fever occurrence

	Crude OR (95% CI)	p	Adjusted OR (95% CI)	p
Antimalaria drugs				
ACT	1		1	
Quinine	47.31 (10.64–210.3)	< 0.001	57.33 (11.65–282.08)	< 0.001
Genotypes				
MBL_2_*A/A	1		1	
MBL2*A/B or A/C	0.21 (0.06–0.78)	0.019	0.09 (0.01–0.63)	0.015
MBL2*BC or C/C	0.58 (0.24–1.43)	0.237	0.71 (0.19–2.66)	0.608
Status G6PD				
Normal	1		1	
Deficient	0.35 (0.14–0.54)	0.017	0.70 (0.19–2.56)	0.586
Parasitaemia				
< 1000 trophozoites/µl	1		1	
> 1000 trophozoites/µl	3.3 (1.40–7.69)	0.005	5.76 (1.79–18.55)	0.003

The association between alleles and genotypes, and each of the 2 clinical groups was also assessed. The A allele was the most common in BWF group as well as in the UM group with allele frequency of 76.7 and 61.0%, respectively, and the difference was not statistically significant, OR: 2.67 (0.87–8.29 and *p *= 0.079 (Table 3). Conversely, the C allele frequency was 0.186 and 0.291 in BWF and UM groups, respectively, and the difference was not statistically significant (*p *= 0.853). Not a single D allele was encountered in the present study population (Table 3). Regarding the genotypes; the proportion of homozygote’s AA was higher in the BWF group (72.0%) compared to the UM (50.0%). Conversely, the *00* genotype was proportionately more frequent in the UM (27.9%) than in BWF (18.6%) (Table 3). A0 genotype is significantly over-represented in UM population compared to BWF patients, OR: 0.21 (0.06–0.78) with p = 0.019 (Table 3).

**Table 3:** Alleles and genotypes Frequencies for the 3 polymorphisms in the Exon 1

	Blackwater fever	Uncomplicated malaria	Total	Crude OR 95% CI	*p* values
Alleles	n (freq)	n (freq)	n (freq)		
A	66 (0.767)	105 (0.610)	171 (0.663)	2.67 (0.86–8.29)	0.079
B	4 (0.046)	17 (0.098)	21 (0.081)	1	
C	16 (0.186)	50 (0.291)	66 (0.256)	1.35 (0.41–5.30)	0.858
D	0 (0.00)	0 (0.00)	0 (0.00)	–	
Total allele freq	86 (1.00)	172 (1.00)	258 (1.00)	–	
Genotypes	n (freq)	n (freq)	n (freq)		
AA	31 (0.721)	43 (0.500)	74 (0.574)	1	
A0	4 (0.093)	19 (0.221)	23 (0.178)	0.21 (0.06–0.78)	0.019
AB	1	7	8		
AC	3	12	15		
AD	0	0	0		
00	8 (0.186)	24 (0.279)	32 (0.248)	0.58 (0.24–1.43)	0.237
BC	3	10	13		
CC	5	14	19		
BD	0	0	0		
CD	0	0	0		
Total genotype freq	43 (1.00)	86 (1.00)	129 (1.00)		

Nine haplotypes were encountered in this study cohort, including 3 high MBL expression haplotypes and 6 low MBL expression haplotypes (Table 4). The high expression MBL2*LYQA haplotype was the most prevalent haplotype in BWF as well as in UM, with 46.3 and 39.5%, respectively. Low MBL expression haplotypes were; MBL2*HYPB; MBL2*HYPC; MBL2*LYQC (Y16578); MBL2*LYPC, MBL2*LYPB (Y16579); MBL2*LXPA and were not significant. Only MBL2*LYQA haplotype was consistently over-represented in UM group, but not significantly (Table 4). None of the groups deviated from the Hardy–Weinberg expectations [40] as showed in Table 3.

**Table 4:** MBL2 haplotypes (promoter region and exon1) and risk assessment

Haplotypes	BWF, n (%)	UM, n (%)	Total n (%)	p
High MBL expression				
MBL2*LYQA (Y16576)	20 (46.1)	24 (39.5)	54 (41.9)	-NS
MBL2*HYPA (Y16581)	6 (14)	11 (12.8)	17 (13.2)	NS
MBL2*LYPA (Y16577)	3 (7.0)	2 (2.3)	5 (3.9)	NS
Low MBL expression				
MBL2*HYPB	0 (0.0)	1 (1.2)	1 (0.8)	NS
MBL2*HYPC	1 (2.3)	1 (1.2)	2 (1.5)	NS
MBL2*LYQC (Y16578)	5 (11.6)	20 (23.3)	25 (19.4)	NS
MBL2*LYPC	0 (0.0)	1 (1.2)	1 (0.8)	NS
MBL2*LYPB (Y16579)	1 (2.3)	3 (3.5)	4 (3.1)	NS
MBL2*LXPA	7 (16.3)	13 (15.1)	20 (15.5)	NS
Total	43 (100)	86 (100)	129 (100)	

## Discussion

The present study investigated whether some alleles, genotypes or haplotypes were significantly over-represented or under-represented in patients with BWF compared to those with UM. A cohort of 129 patients was recruited from 4 hospitals across Kinshasa. Only a few of them were within higher risk group to develop severe malaria, meaning below 5 years of age, as described in many studies. However, the majority of recruited patients was at risk for BWF as this form of malaria is mostly observed in older children and adults [12, 13, 17, 21, 40–45].

### Allele frequency

MBL2*A allele was the most common allele within the 2 groups compared to each of the derived alleles individually. However, when considered together, null alleles (allele 0) were more frequent among patients with UM compared to those with BWF, with allele frequencies of 0.39 and 0.233, respectively. 0 includes B, C and D alleles (Table 3). MBL2*C was the most frequent in both groups. Bellamy et al. [46] reported also a higher frequency of the MBL2*C in in the population of The Gambia. Compared to the other null alleles, the *MBL*2*C has been demonstrated to be extremely common in sub-Saharan Africans with a population frequency of 0.30, whereas the MBL2*B was predominant in Europeans, in Asians and in indigenous people of South America with population frequencies of 0.13, 0.20 and 0.50, respectively [3, 5, 46]. None of the alleles observed in the study population presented a significant preferential distribution between the 2 groups.

It has been hypothesized that *B, *C and *D alleles are positively selected in order to reduce susceptibility or mortality due to certain infectious diseases [5, 24, 34]. This study did not identify the MBL2*D allele within the 2 groups. This allele has been detected with frequencies up to 0.05 in the northeast of Africa, in Europe and India [3, 10]. Hence, the absence of the MBL2*D may simply indicate a low admixture with European and Indian in the Congolese population examined in this study [47–49].

### *MBL2* genotypes and BWF

#### MBL might protect against severe disease forms but not against BWF

Multiple genetic epidemiological studies reported that the presence *MBL2* derived alleles and genotypes are associated with an increased risk to infections [4, 28, 29, 32, 50] and might be considered as a prognostic marker in various infectious conditions [29, 32, 51, 52]. Functional studies showed that heterozygotes for a *MBL2* variant produces low concentration of MBL protein and this may hamper the phagocytosis of bacteria or parasites, thereby allowing the replication of the pathogen [24, 28, 48, 53, 54]. Based on this group of studies, one would expect individuals with ancestral *MBL2* AA alleles to be protected against BWF, a severe phenotype. Unlike in other severe forms of malaria, such association was not observed in this study.

#### Homozygotes for ancestral MBL2 alleles are at higher risk for BWF

The other wildly supported theory is that low levels of functional MBL may decrease excessive activation of the immune response and enhance survival in some patients [5, 34, 35]. Therefore, low levels of functional MBL protects against severe complications triggered by the host immune response. In this study individuals with AA genotype had higher risk for BWF as compared to A0 genotype, which is consistent with the second theory. This observation and the previously reported elevated levels of malaria IgG in BWF suggests that BWF might be caused mainly by excessive activation of the immune response. The current results do not formally exclude the role of MBL deficiency in the occurrence of BWF since *00* individuals presented with intermediate risk for BWF as compared to A0.

### Haplotypes

The present study revealed 9 haplotypes, including 3 high MBL expression haplotypes (Table 4) and 6 low expression haplotypes. The LYQA haplotype was the most prevalent haplotype both in BWF and UM group with, respectively, 45.5 and 39.5%, followed by MBL2⃰ LYQC in UM population with 23.3%. In Gabon, Boldt et al. defined 14 new haplotypes and reported that *MBL*2*LYQC, MBL2*LYQA and MBL2*LYPA were the most prevalent haplotypes in the children population [55]. A new haplotype HYPC only described in Zimbabwe individuals was observed in 2 patients: one BWF and one UM. A study in India reported that the MBL2*LYPA haplotypes confers protection, whereas MBL2*LXPA increases the malaria risk. These findings in Indian populations demonstrate that *MBL2* functional variants are strongly associated with malaria and infection severity [10]. However, no significant association was find between BWF and a particular haplotype.

### Parasitaemia and BWF

Lower parasitaemia was observed in BWF patients. Considering that quinine intake offers a strong clearance of parasite, low parasitaemia observed in BWF may be secondary to the quinine intake that triggers BWF occurrence. In that prospect, the time between quinine intake and the occurrence of BWF may influence parasitaemia. However, this timing remains unclear since reported time-lapses range from 12 h to multiple days after treatment [56–58]. Another reason for low parasitaemia in BWF could be the activity of the immune system in AA individuals. The active immune response would provide a good clearance of parasite and accessorily cause BWF. Further studies may be needed to investigate this hypothesis.

### Limitations of the study

The major limitation of this study is the small sample size. Although BWF is rare in the study setting, the small sample size may have influenced the statistical calculations. Another limitation was that the investigation of G6PD polymorphisms, and the complement activation and MBL protein were not measured. In addition, no data exist in the community about the frequency of *MBL2* polymorphism in the country. Despite these limitations, these data provide insights into the relationship between MBL protein level/activity and BWF, and could form a basis for further studies in a large Congolese population.

## Conclusion

This pilot study is a preliminary research on *MBL2* gene and infectious diseases in DRC. The result shows a higher risk for BWF in AA. This suggests that future studies on BWF should further investigate the contribution of a strong immune response to the occurrence of BWF.

## Data Availability

The datasets used and/or analysed during the current study are available from the corresponding author on reasonable request.

## Abbreviations

DRC: Democratic Republic of Congo
UM: uncomplicated malaria
BWF: blackwater fever
MBL: Mannose Binding Lectin protein
*MBL2*: Mannose Binding Lectin gene

## References

[1] Naito H, Ikeda A, Hasegawa K, Oka S, Uemura K, Kawasaki N (1999). Characterization of human serum mannan-binding protein promoter. J Biochem.

[2] Giubergia V, Salim M, Fraga J, Castiglioni N, Sen L, Castanos C (2015). Post-infectious bronchiolitis obliterans and mannose-binding lectin insufficiency in Argentinean children. Respirology.

[3] Madsen HO, Garred P, Kurtzhals JA, Lamm LU, Ryder LP, Thiel S (1994). A new frequent allele is the missing link in the structural polymorphism of the human mannan-binding protein. Immunogenetics.

[4] Sumiya M, Super M, Tabona P, Levinsky RJ, Arai T, Turner MW (1991). Molecular basis of opsonic defect in immunodeficient children. Lancet.

[5] Lipscombe RJ, Sumiya M, Hill AV, Lau YL, Levinsky RJ, Summerfield JA (1992). High frequencies in African and non-African populations of independent mutations in the mannose binding protein gene. Hum Mol Genet.

[6] Garred P, Larsen F, Seyfarth J, Fujita R, Madsen HO (2006). Mannose-binding lectin and its genetic variants. Genes Immun.

[7] Antonarakis SE (1998). Recommendations for a nomenclature system for human gene mutations. Nomenclature Working Group. Hum Mutat..

[8] Eisen DP, Minchinton RM (2003). Impact of mannose-binding lectin on susceptibility to infectious diseases. Clin Infect Dis.

[9] Madsen HO, Garred P, Thiel S, Kurtzhals JA, Lamm LU, Ryder LP (1995). Interplay between promoter and structural gene variants control basal serum level of mannan-binding protein. J Immunol..

[10] Jha AN, Sundaravadivel P, Singh VK, Pati SS, Patra PK, Kremsner PG (2014). MBL2 variations and malaria susceptibility in Indian populations. Infect Immun.

[11] Mhandire K, Pharo G, Kandawasvika GQ, Duri K, Swart M, Stray-Pedersen B (2014). How does mother-to-child transmission of HIV differ among African populations? Lessons from MBL2 genetic variation in Zimbabweans. OMICS.

[12] Bodi JM, Nsibu CN, Longenge RL, Aloni MN, Akilimali PZ, Tshibassu PM (2013). Blackwater fever in Congolese children: a report of clinical, laboratory features and risk factors. Malar J..

[13] Delacollette C, Taelman H, Wery M (1995). An etiologic study of hemoglobinuria and blackwater fever in the Kivu Mountains, Zaire. Ann Soc Belg Med Trop..

[14] Ministère du Plan et Suivi de la Mise en Oeuvre de la Révolution et de la Modernité Modernité, Ministère de la Santé Publique. Enquête Démographique et de la Santé. République Démocratique du Congo, 2013.

[15] Mokoli MV, Nseka MN, Lepira FB, Sumaili EK, Bukabau J (2007). Profil clinico-bio-morphologique et évolutif de l’insuffisance rénale aiguë aux Cliniques Universitaires de Kinshasa. Annales Africaines de Médecine..

[16] Nebie I, Diarra A, Ouedraogo A, Soulama I, Bougouma EC, Tiono AB (2008). Humoral responses to Plasmodium falciparum blood-stage antigens and association with incidence of clinical malaria in children living in an area of seasonal malaria transmission in Burkina Faso, West Africa. Infect Immun..

[17] Aloni MN, Nsibu CN, Meeko-Mimaniye M, Ekulu PM, Bodi JM (2012). Acute renal failure in Congolese children: a tertiary institution experience. Acta Paediatr.

[18] Assounga AG, Assambo-Kieli C, Mafoua A, Moyen G, Nzingoula S (2000). Etiology and outcome of acute renal failure in children in congo-brazzaville. Saudi J Kidney Dis Transpl..

[19] Bruneel F, Gachot B, Wolff M, Bedos JP, Regnier B, Danis M (2002). Blackwater fever. Presse Med.

[20] Das BS (2008). Renal failure in malaria. J Vector Borne Dis..

[21] Kunuanunua TS, Nsibu CN, Gini-Ehungu JL, Bodi JM, Ekulu PM, Situakibanza H (2013). Acute renal failure and severe malaria in Congolese children living in Kinshasa, Democratic Republic of Congo. Nephrol Ther..

[22] Daubrey-Potey T, Die-Kacou H, Kamagate M, Vamy M, Balayssac E, Yavo JC (2004). Blackwater fever during antimalarial treatment in Abidjan (West Africa): report of 41 cases. Bull Soc Pathol Exot.

[23] Garred P, Nielsen MA, Kurtzhals JA, Malhotra R, Madsen HO, Goka BQ (2003). Mannose-binding lectin is a disease modifier in clinical malaria and may function as opsonin for Plasmodium falciparum-infected erythrocytes. Infect Immun.

[24] Madsen HO, Satz ML, Hogh B, Svejgaard A, Garred P (1998). Different molecular events result in low protein levels of mannan-binding lectin in populations from southeast Africa and South America. J Immunol..

[25] WHO Group (2015). Guidelines for the Treatment of Malaria.

[26] Steffensen R, Thiel S, Varming K, Jersild C, Jensenius JC (2000). Detection of structural gene mutations and promoter polymorphisms in the mannan-binding lectin (MBL) gene by polymerase chain reaction with sequence-specific primers. J Immunol Methods.

[27] Casanova JL, Abel L (2004). Human Mannose-binding Lectin in Immunity: friend, Foe, or Both?. J Exp Med.

[28] Garred P, Madsen HO, Balslev U, Hofmann B, Pedersen C, Gerstoft J (1997). Susceptibility to HIV infection and progression of AIDS in relation to variant alleles of mannose-binding lectin. Lancet.

[29] Garred P, Madsen HO, Hofmann B, Svejgaard A (1995). Increased frequency of homozygosity of abnormal mannan-binding-protein alleles in patients with suspected immunodeficiency. Lancet.

[30] Gordon AC, Waheed U, Hansen TK, Hitman GA, Garrard CS, Turner MW (2006). Mannose-binding lectin polymorphisms in severe sepsis: relationship to levels, incidence, and outcome. Shock..

[31] Sullivan KE, Wooten C, Goldman D, Petri M (1996). Mannose-binding protein genetic polymorphisms in black patients with systemic lupus erythematosus. Arthritis Rheum.

[32] Summerfield JA, Ryder S, Sumiya M, Thursz M, Gorchein A, Monteil MA (1995). Mannose binding protein gene mutations associated with unusual and severe infections in adults. Lancet.

[33] Summerfield JA, Sumiya M, Levin M, Turner MW (1997). Association of mutations in mannose binding protein gene with childhood infection in consecutive hospital series. BMJ.

[34] Garred P, Thiel S, Madsen HO, Ryder LP, Jensenius JC, Svejgaard A (1992). Gene frequency and partial protein characterization of an allelic variant of mannan binding protein associated with low serum concentrations. Clin Exp Immunol.

[35] Takahashi K, Gordon J, Liu H, Sastry KN, Epstein JE, Motwani M (2002). Lack of mannose-binding lectin-A enhances survival in a mouse model of acute septic peritonitis. Microbes Infect.

[36] Bodi J, Nsibu C, Longenge R, Aloni M, Akilimali P, Tshibassu P (2018). High IgG1 malaria antibodies level in children is a possible risk factor of blackwater fever: a case-control study. Pediatr Health Res..

[37] Monsey L, Best LG, Zhu J, DeCroo S, Anderson MZ (2019). The association of mannose binding lectin genotype and immune response to *Chlamydia pneumoniae*: the Strong Heart Study. PLoS ONE.

[38] Assoumanou MG, Akpona SA (2011). Dosage de l’hémoglobine urinaire par un réactif 3,3′ diméthylbenzidine: mise au point technique. Int J Biol Chem Sci..

[39] Dean FB, Nelson JR, Giesler TL, Lasken RS (2001). Rapid amplification of plasmid and phage DNA using Phi 29 DNA polymerase and multiply-primed rolling circle amplification. Genome Res.

[40] Gobbi F, Audagnotto S, Trentini L, Nkurunziza I, Corachan M, Di Perri G (2005). Blackwater fever in children, Burundi. Emerg Infect Dis..

[41] Bouldouyre MA, Dia D, Carmoi T, Fall KB, Chevalier B, Debonne JM (2006). A mild blackwater fever. Med Mal Infect..

[42] Khandelwal V, Udawat H, Kumhar MR, Goyal RK (2001). Blackwater fever treated with artemether. J Assoc Physicians India.

[43] Rogier C, Imbert P, Tall A, Sokhna C, Spiegel A, Trape JF (2003). Epidemiological and clinical aspects of blackwater fever among African children suffering frequent malaria attacks. Trans R Soc Trop Med Hyg.

[44] Tran TH, Day NP, Ly VC, Nguyen TH, Pham PL, Nguyen HP (1996). Blackwater fever in southern Vietnam: a prospective descriptive study of 50 cases. Clin Infect Dis.

[45] WHO (2012). Malaria Policy Advisory Committee to the WHO: conclusions and recommendations of September 2012 meeting. Malar J..

[46] Bellamy R, Ruwende C, McAdam KP, Thursz M, Sumiya M, Summerfield J (1998). Mannose binding protein deficiency is not associated with malaria, hepatitis B carriage nor tuberculosis in Africans. QJM.

[47] Bernig T, Taylor JG, Foster CB, Staats B, Yeager M, Chanock SJ (2004). Sequence analysis of the mannose-binding lectin (MBL2) gene reveals a high degree of heterozygosity with evidence of selection. Genes Immun.

[48] Boldt AB, Messias-Reason IJ, Meyer D, Schrago CG, Lang F, Lell B (2010). Phylogenetic nomenclature and evolution of mannose-binding lectin (MBL2) haplotypes. BMC Genet.

[49] Verdu P, Barreiro LB, Patin E, Gessain A, Cassar O, Kidd JR (2006). Evolutionary insights into the high worldwide prevalence of MBL2 deficiency alleles. Hum Mol Genet.

[50] Guo SW, Thompson EA (1992). Performing the exact test of Hardy–Weinberg proportion for multiple alleles. Biometrics.

[51] Garred P, Harboe M, Oettinger T, Koch C, Svejgaard A (1994). Dual role of mannan-binding protein in infections: another case of heterosis?. Eur J Immunogenet.

[52] Garred P, Madsen HO, Halberg P, Petersen J, Kronborg G, Svejgaard A (1999). Mannose-binding lectin polymorphisms and susceptibility to infection in systemic lupus erythematosus. Arthritis Rheum.

[53] Hoal-Van Helden EG, Epstein J, Victor TC, Hon D, Lewis LA, Beyers N (1999). Mannose-binding protein B allele confers protection against tuberculous meningitis. Pediatr Res.

[54] Santos IK, Costa CH, Krieger H, Feitosa MF, Zurakowski D, Fardin B (2001). Mannan-binding lectin enhances susceptibility to visceral leishmaniasis. Infect Immun.

[55] Boldt AB, Petzl-Erler ML (2002). A new strategy for mannose-binding lectin gene haplotyping. Hum Mutat.

[56] Sher A, Ed. Hemoglobinuria (Black Water Fever) in severe falciparum malaria—a case report. In: 17th International Congress on Infectious Diseases; 2016.

[57] Lon C, Spring M, Sok S, Chann S, Bun R, Ittiverakul M (2014). Blackwater fever in an uncomplicated *Plasmodium falciparum* patient treated with dihydroartemisinin–piperaquine. Malar J..

[58] Rodriguez-Valero N, Castro P, Martinez G, Marco Hernandez J, Fernandez S, Gascon J (2018). Blackwater fever in a non-immune patient with *Plasmodium falciparum* malaria after intravenous artesunate. J Travel Med..

